# 基于原位生长法制备球状共价有机框架涂层并用于开管毛细管电色谱分离中性分析物

**DOI:** 10.3724/SP.J.1123.2026.04009

**Published:** 2026-11-08

**Authors:** Xiao NIU, Yang ZHANG, Shengda QI, Hongli CHEN

**Affiliations:** 兰州大学化学化工学院，甘肃 兰州 730000; College of Chemistry and Chemical Engineering，Lanzhou University，Lanzhou 730000，China

**Keywords:** 共价有机框架, 原位生长, 开管毛细管电色谱, 邻苯二甲酸酯类, covalent organic frameworks （COFs）, in-situ growth, open-tubular capillary electrochromatography （OT-CEC）, phthalate esters

## Abstract

共价有机框架（COFs）材料因其结构可调、孔隙丰富、比表面积大等优势，已成为一类性能优异的色谱固定相。本文采用室温原位生长法在毛细管内壁成功涂敷了一层球状COF（TPB-TP COF），并将其应用于开管毛细管电色谱（OT-CEC）分离分析中。研究结果表明，TPB-TP COF纳米晶体能够牢固地固定于毛细管内壁，运行200次后，涂层表面结构基本保持完整。与未修饰的裸毛细管相比，该涂层柱对4组中性分析物表现出更优异的分离能力与高分辨率，且具备良好的重复性和稳定性。保留行为分析表明，分析物的洗脱顺序与其log *K*_ow_值具有一定相关性，说明疏水作用可能是影响分离过程的重要因素；同时，TPB-TP COF中丰富的芳香结构可能通过*π*-*π*相互作用对芳香类分析物的保留和选择性产生协同影响。该方法已成功应用于加标黄河水样中邻苯二甲酸酯类化合物的测定，加标回收率为94.9%~108.9%。上述结果表明，TPB-TP COF是一种有效的OT-CEC固定相材料，在中性有机小分子分离分析中具有潜在应用价值。

## Abstract (en)

A spherical covalent organic framework （TPB-TP COF） was successfully prepared via a facile and room-temperature in-situ growth method and applied as stationary phase in open-tubular capillary electrochromatography （OT-CEC） for the separation of neutral analytes. The TPB-TP COF was synthesized through the Schiff-base condensation of 1，3，5-tris（4-aminophenyl）benzene （TPB） and terephthalaldehyde （TP）. The inner wall of the fused-silica capillary was first pretreated with NaOH and HCl，followed by modification with 3-（2，3-epoxypropoxy）propyltrimethoxysilane （GLYMO） to provide active epoxy groups for the covalent attachment of COF nanocrystals. Comprehensive characterizations confirmed the successful construction of the coating. Powder X-ray diffraction （PXRD） patterns exhibited high crystallinity consistent with literature reports. Fourier transform infrared （FT-IR） spectra showed a characteristic stretching vibration of the imine bond （-C=N） at 1 615 cm^-1^，confirming the framework formation. Scanning electron microscopy （SEM） and transmission electron microscopy （TEM） revealed a uniform spherical morphology. Nitrogen adsorption-desorption isotherms indicated a microporous structure with a pore size of 1.47 nm and a BET surface area of 20 m^2^/g. Thermogravimetric （TG） analysis demonstrated excellent thermal stability up to 400 ℃. The electroosmotic flow （EOF） properties were systematically investigated，showing that the TPB-TP COF coating effectively masked the silanol groups on the capillary wall，resulting in a significantly lower and more stable EOF compared to the bare capillary. Notably，the unique structural features of TPB-TP COF provided significant kinetic advantages in OT-CEC. Unlike conventional COFs that pursue extremely high surface areas，the modest BET surface area of the spherical TPB-TP COF featured rapid mass transfer kinetics by minimizing solute entrapment in deep and complex pores. The dynamic mass transfer optimization strategy effectively suppressed peak broadening and enhanced separation efficiency. The separation performance was evaluated using four groups of neutral analytes，including substituted benzenes （toluene，*p*-xylene，1，3，5-trimethylbenzene），halobenzenes （fluorobenzene，bromobenzene，*o*-dibromobenzene，1-bromo-4-iodobenzene），and two sets of phthalate esters （PAEs）. After optimizing parameters such as acetonitrile content，buffer pH，and borate concentration，the coated capillary achieved baseline separation for all analytes. The elution order followed the increasing order of their octanol-water partition coefficients （log *K*_ow_），indicating a typical reversed-phase chromatographic mechanism dominated by strong hydrophobic interactions and *π*-*π* stacking interactions between the aromatic framework and the analytes. The stability and reproducibility of the TPB-TP COF coated capillary were rigorously validated. The relative standard deviations （RSDs） for intra-day，inter-day，and column-to-column migration times and peak areas were all below 10%. Furthermore，the coating maintained its structural integrity and separation efficiency even after 200 consecutive runs，demonstrating high mechanical and chemical robustness. Finally，the proposed OT-CEC method was applied to determine PAEs in Yellow River water samples. The method exhibited wide linear ranges （2.0-100.0 μg/mL and 5.0-100.0 μg/mL） with correlation coefficients （*R* ^2^） higher than 0.987 7. The limits of detection （LODs） and quantification （LOQs） were 0.14-0.37 μg/mL and 0.47-1.22 μg/mL，respectively. The spiked recoveries ranged from 94.9% to 108.9% with RSDs of 1.0%-11.3%，confirming the reliability and practical utility of the method. These results highlight the immense potential of TPB-TP COF as a high-performance stationary phase for advanced chromatographic separations.

毛细管电色谱（capillary electrochromatography，CEC）具有分离效率高、选择性好等优点^［1］^。固定相的选择及色谱柱制备技术的发展对于改善CEC的分离性能至关重要。CEC毛细管柱主要分为3种类型：填充柱、整体柱和开管柱（open tubular column）。作为一种重要的CEC柱，开管柱具有制备工艺简单、改性容易、稳定性好等优点，引起了许多研究者的关注^［2］^。随着开管毛细管电色谱（OT-CEC）的不断发展，各种纳米材料被引入作为新型固定相，以提高OT-CEC的分离效率，如氧化石墨烯^［3］^、金属有机框架（MOFs）^［4，5］^、生物材料^［6］^和共价有机框架（COFs）^［7，8］^等。到目前为止，开管毛细管柱的制备方法主要有物理涂覆法和共价键合法^［9］^。虽然物理涂覆法操作简单且直接，但因其制备的涂层不均匀且易脱落，导致色谱固定相寿命较短和分离效果不佳，限制了其在OT-CEC中的进一步应用。因此，发展性能更为优异的共价键合法制备开管毛细管柱的新型固定相材料十分必要。

COFs是一类新兴的结晶多孔材料，由有机构建块通过共价键^［10］^构成，具有结构多样性、高比表面积和孔隙率、良好的稳定性和低密度等独特特性，因此在催化^［11，12］^、传感和检测^［13］^、光电子学^［14］^、药物传递^［15-17］^、能量存储^［18，19］^和分离^［20-24］^中具有广泛应用。随着COFs的发展，研究人员一直尝试改进COFs的合成方法以制备不同连接键的COFs。其中，亚胺类COFs因其单体易得、合成方式温和而备受关注。

然而，在OT-CEC分离中，固定相的孔结构、表面性质和形貌均可能影响溶质传质及峰形。对于部分高比表面积、多孔COFs材料而言，复杂孔道结构可能增加溶质在固定相中的扩散路径，从而在一定程度上影响传质效率和柱效。因此，构建形貌规整、传质路径较短且具有适当相互作用位点的COF涂层固定相，对提高OT-CEC分离性能具有重要意义。本研究通过原位生长法成功构建了亚胺键（-C=N）连接的球形TPB-TP COF涂层毛细管柱。该材料在结构设计与分离机制上表现出显著特色，首先，TPB-TP COF具有规则的纳米球形结构（平均直径约250 nm）与适中的比表面积（20 m^2^/g），有效缩短了溶质在孔道中的滞留时间，通过消除扩散迟滞显著降低了传质阻力，从而实现了高柱效分离；与此同时，TPB-TP COF骨架中含有丰富的芳香环和-C=N，可为中性分析物提供疏水作用、*π*-*π*相互作用以及可能的偶极相互作用位点。实验结果表明，该涂层柱对4组中性分析物均呈现出优异的分离性能。

## 1 实验部分

### 1.1 仪器、试剂与材料

JEM-2100透射电子显微镜（TEM）和JSM-6701F扫描电子显微镜（SEM）购自日本电子株式会社（JEOL）；90Plus PALS Zeta电位及粒度分析仪购自美国布鲁克海文仪器公司（Brookhaven）；Nicolet iS50傅里叶变换红外光谱仪（FT-IR）购自美国赛默飞世尔科技公司（Thermo Scientific）；STA PT1600综合热分析仪购自德国Linseis公司；Tristar3020及ASAP2020M全自动比表面积物理吸附仪购自美国麦克仪器公司（Micromeritics）。Empyrean粉末X射线衍射仪（PXRD）购自荷兰帕纳科公司（PANalytical），辐射源为Cu Kα 射线（λ=1.540 59 Å）；OT-CEC实验在自搭的毛细管电泳仪上完成，高压电源购自东文高压电源有限公司，配备有CoMetro 2600紫外-可见检测器和万象V2008MAX色谱工作站系统。

对苯二甲醛（TP，纯度>98%）购自吉林省研伸科技有限公司，对二甲苯（*p*-xylene，纯度>99%）、邻苯二甲酸二环己基酯（DCHP，纯度>98%）、邻苯二甲酸二乙酯（DEP，纯度>98%）和邻苯二甲酸二苄酯（DBZP，纯度>98%）购自上海安耐吉化学有限公司；3-（2，3-环氧丙氧）丙基三甲氧基硅烷（GLYMO）、1，3，5-三甲苯（1，3，5-trimethylbenzene，纯度>96%）和氟苯（fluorobenzene，纯度>99%）购自东京化成工业株式会社（日本）；溴苯（bromobenzene，纯度>97%）、邻二溴苯（*o*-dibromobenzene，纯度>99%）、4-溴碘苯（1-bromo-4-iodobenzene，纯度>99%）和邻苯二甲酸二异丙酯（DPrP，纯度>97%）购自上海梯希爱化成工业发展有限公司；邻苯二甲酸二甲氧基乙酯（DMEP，纯度>99%）、邻苯二甲酸二异丁酯（DIBP，纯度>98%）、邻苯二甲酸二辛酯（DNOP，纯度>98%）和邻苯二甲酸二烯丙酯（DAP，纯度>98%）购自上海阿达玛斯试剂有限公司；甲苯（toluene，纯度>98%）购自上海阿拉丁试剂有限公司，1，3，5-三（4-氨基苯基）苯（TPB，纯度>98%）购自吉林省科研器材有限公司；石英毛细管（内径75 μm，外径375 μm）购自河北永年锐沣色谱器件有限公司。

### 1.2 实验过程

#### 1.2.1 TPB-TP COF的合成

按照文献［^23^］的方法合成TPB-TP COF，具体过程如图1所示：将TPB（0.04 mmol）和TP（0.06 mmol）加入10 mL离心管中，加入5 mL乙腈，超声溶解得到澄清溶液，而后加入0.4 mL 12 mol/L乙酸，涡旋10 s，室温下反应3 d。反应结束后，用乙腈和乙醇依次离心洗涤3次得到黄色沉淀，于60 ℃下真空干燥24 h，得到黄色粉末TPB-TP COF。

**图1:**
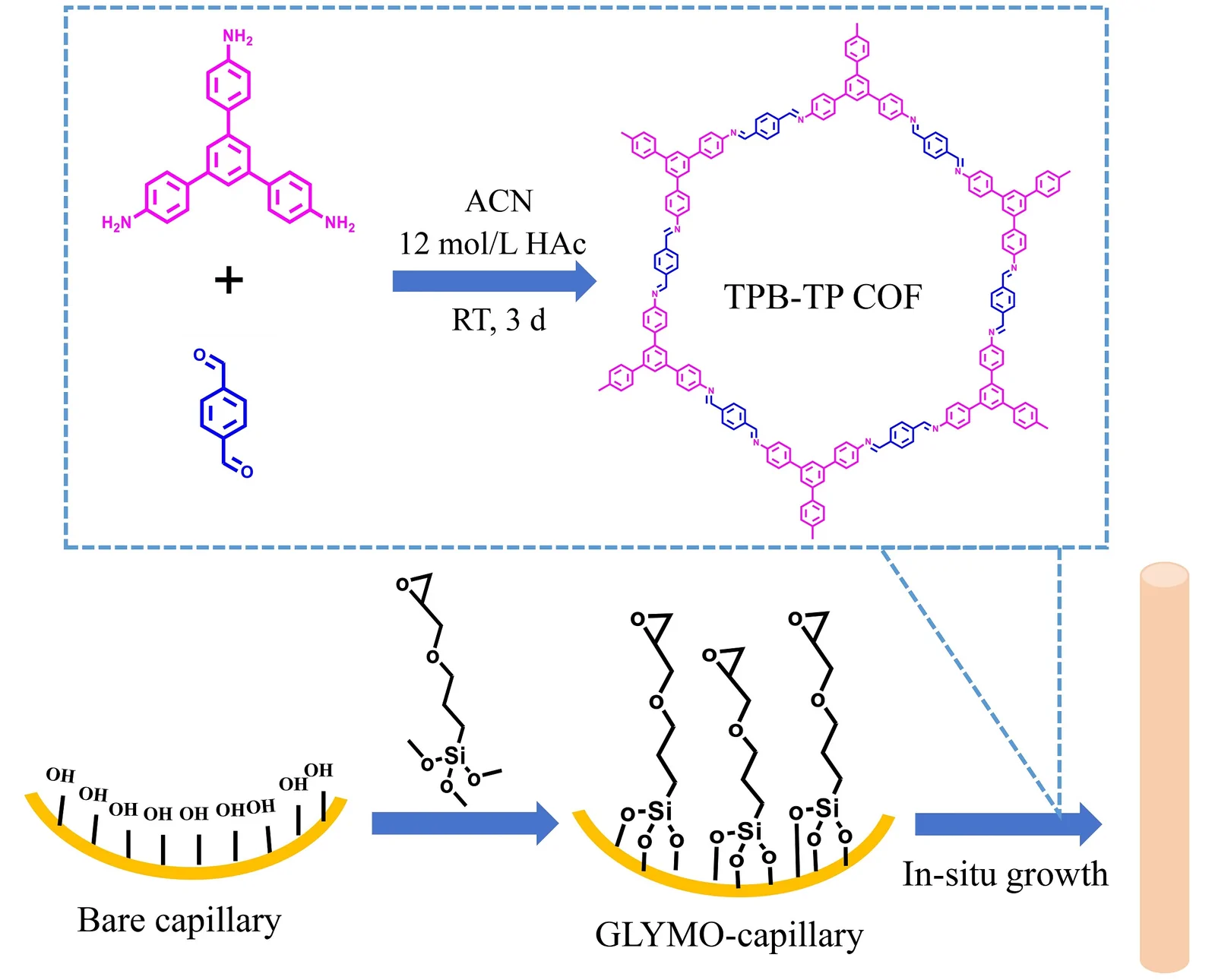
TPB-TP COF涂层毛细管的制备示意图 TPB： 1，3，5-tris（4-aminophenyl）benzene； TP： terephthalaldehyde； COF： covalent organic framework； ACN： acetonitrile； HAc： acetic acid； RT： room temperature； GLYMO： 3-glycidyloxypropyltrimethoxysilane.

#### 1.2.2 TPB-TP COF涂层毛细管的制备

首先，将熔融石英毛细管依次用甲醇、超纯水和1.0 mol/L NaOH溶液分别冲洗10、10和30 min。冲洗完毕后，密封毛细管两端，将其置于100 ℃烘箱中加热2 h。待冷却后，依次用0.5 mol/L HCl（30 min）、超纯水（10 min）、甲醇（10 min）和丙酮（10 min）对毛细管进行冲洗，经氮气（N_2_）吹干后，置于150 ℃烘箱中反应2 h。待毛细管冷却至室温后，将5% GLYMO甲苯溶液注入预处理后的毛细管中并保留15 min。密封毛细管两端，于100 ℃烘箱中反应8 h。反应结束后，依次用甲苯、丙酮和甲醇分别冲洗毛细管10 min以去除残留物，最后用N_2_吹干，制得GLYMO改性的毛细管。

随后，进行COF涂层的制备：将0.04 mmol TPB和0.06 mmol TP置于10 mL离心管中，加入5 mL乙腈并超声使其完全溶解。向溶液中加入0.4 mL 12 mol/L乙酸，并在涡旋振荡器上剧烈振荡10 s。立即将混合溶液注入上述 GLYMO 改性的毛细管中，两端用橡胶塞密封，于室温下静置反应72 h。最后，用乙腈冲洗毛细管15 min，制备好的毛细管在N_2_氛围下保存备用。

#### 1.2.3 标准溶液配制

各组分析物的结构式和辛醇-水分配系数（log *K*_ow_）值如图2所示。将邻苯二甲酸酯类分析物溶解于甲醇中，其余分析物溶解于乙腈中，均配制成质量浓度为2.0 mg/mL的标准储备液，置于4 ℃冰箱中避光保存。将上述标准储备液混合并用缓冲溶液稀释得到所需浓度的混合标准工作液。所有样品和缓冲溶液在使用前均经孔径为0.45 μm的纤维素滤膜过滤。

**图2:**
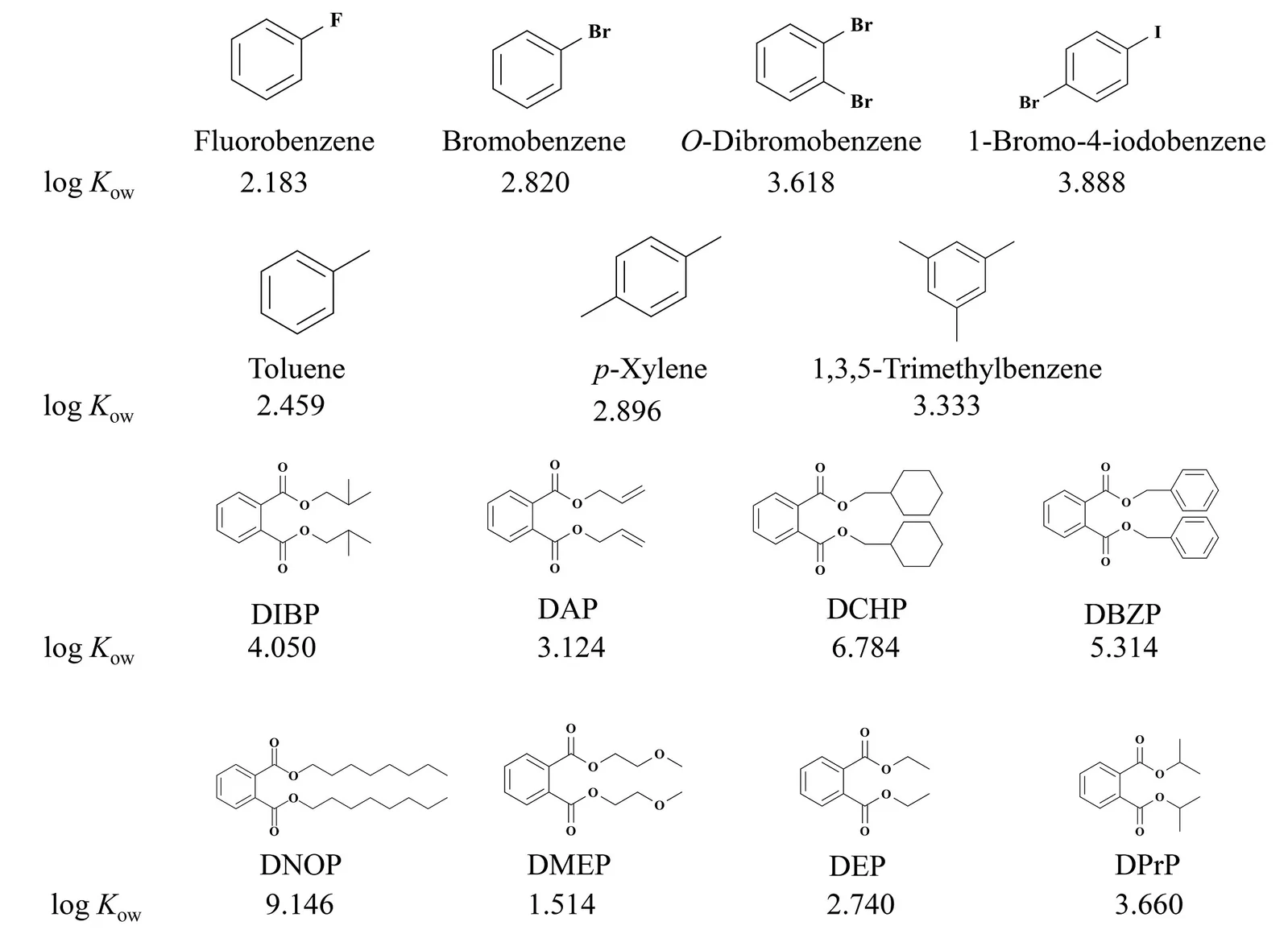
分析物的结构式和log *K*_ow_值 log *K*_ow_： octanol-water partition coefficients； DAP： diallyl phthalate； DIBP： diisobutyl phthalate； DBZP： dibenzyl phthalate； DCHP： dicyclohexyl phthalate； DMEP： bis（2-methoxyethyl）phthalate； DEP： diethyl phthalate； DPrP： diisopropyl phthalate； DNOP： di-*n*-octyl phthalate.

#### 1.2.4 实际样品处理

黄河水样采用10 mL棕色玻璃瓶采集。采样前，玻璃瓶用待采水样润洗两次，并装满密封。样品返回实验室后，立即经0.22 μm微孔滤膜过滤，以去除悬浮颗粒物，滤液置于4 ℃避光保存。

#### 1.2.5 CEC分离条件

在本工作中，毛细管的总长度和有效长度分别为60.0 cm和47.5 cm，内径为75 μm，外径为375 μm。首次使用之前，熔融石英毛细管分别用甲醇（10 min）、超纯水（10 min）、1 mol/L NaOH（30 min）、超纯水（10 min）、1 mol/L HCl（10 min）、超纯水（10 min）和缓冲溶液（5 min）冲洗；涂层毛细管用缓冲溶液冲洗3 min。在两次运行之间，两类毛细管均用超纯水（3 min）和缓冲溶液（3 min）依次冲洗。超声脱气后，以10 cm高度差、3 s的虹吸方式将工作溶液注入毛细管中。分离电压为+20 kV，分析物的检测波长为214 nm。

## 2 结果与讨论

### 2.1 TPB-TP COF及其涂层毛细管的表征

本研究以TPB和TP为单体，在室温条件下反应3 d，成功制备了-C=N键连接的TPB-TP COF。图3a所示的PXRD图谱与文献报道一致^［23］^，表明所合成的COF具有良好的结晶度。图3b是FT-IR图，在1 615 cm^-1^处出现的强吸收峰归属于-C=N的伸缩振动，进一步证明了TPB-TP COF的成功合成。热重分析曲线（图3c）显示，当温度从室温升至400 ℃时，样品质量基本无损失，表明该材料具有优异的热稳定性。N_2_吸附-脱附测试（图3d）呈现典型的Ⅰ型曲线，证实TPB-TP COF内部具有微孔结构，其孔径分布集中于1.47 nm，BET比表面积为20 m^2^/g。此外，由SEM（图3e）和TEM（图3f）的表征结果可见，TPB-TP COF为粒径均一的球形颗粒，平均直径约为250 nm。

**图3:**
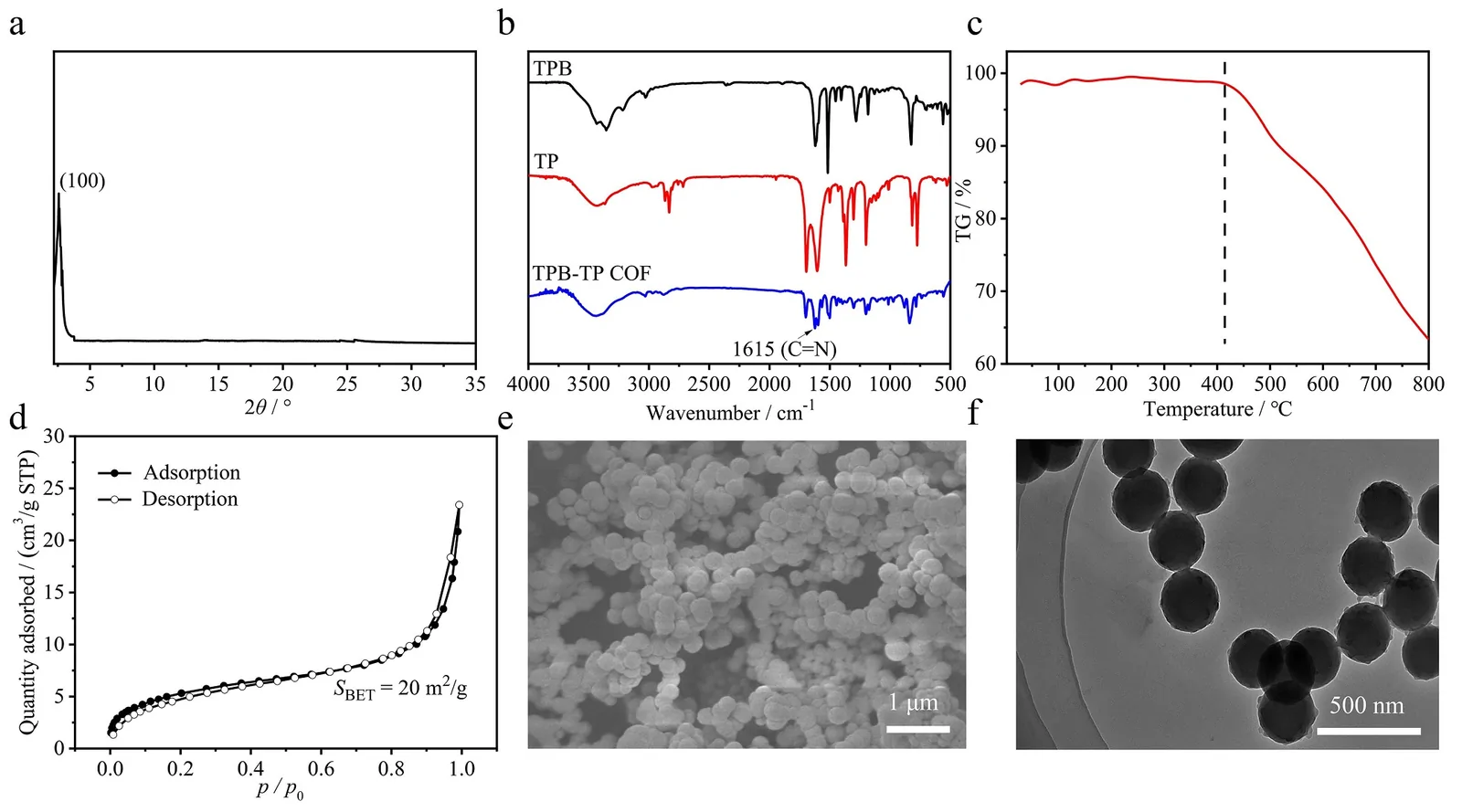
TPB-TP COF的表征：（a）PXRD图谱、（b）FT-IR谱图、（c）热重分析曲线、（d）N_2_吸附-脱附曲线、（e）SEM和（f）TEM图 PXRD： powder X-ray diffraction； FT-IR： Fourier transform infrared spectroscopy； *S*_BET_： BET surface area； SEM： scanning electron microscopy； TEM： transmission electron microscopy.

进而，采用SEM对毛细管内表面的形貌进行了表征。如图4a所示，裸毛细管的内壁非常光滑。相比之下，TPB-TP COF涂层毛细管的内壁（图4b）布满了密集的球形颗粒，其微观形貌与合成的COF粉末（图3e）高度一致。为进一步确证毛细管内壁附着的球形颗粒为TPB-TP COF，选取与毛细管化学组成相似的石英片作为模拟基底，采用完全相同的改性与原位生长工艺进行涂层制备。随后，刮取石英片表面的涂层材料进行FT-IR及SEM表征。FT-IR谱图（图4c）显示，该材料在1 615 cm^-1^处存在明显的-C=N伸缩振动特征峰；同时，SEM图（图4d）表明石英片上涂层材料的尺寸和形貌均与常规室温下制得的TPB-TP COF粉末一致。上述结果充分证明，TPB-TP COF已成功固载于毛细管内壁。

**图4:**
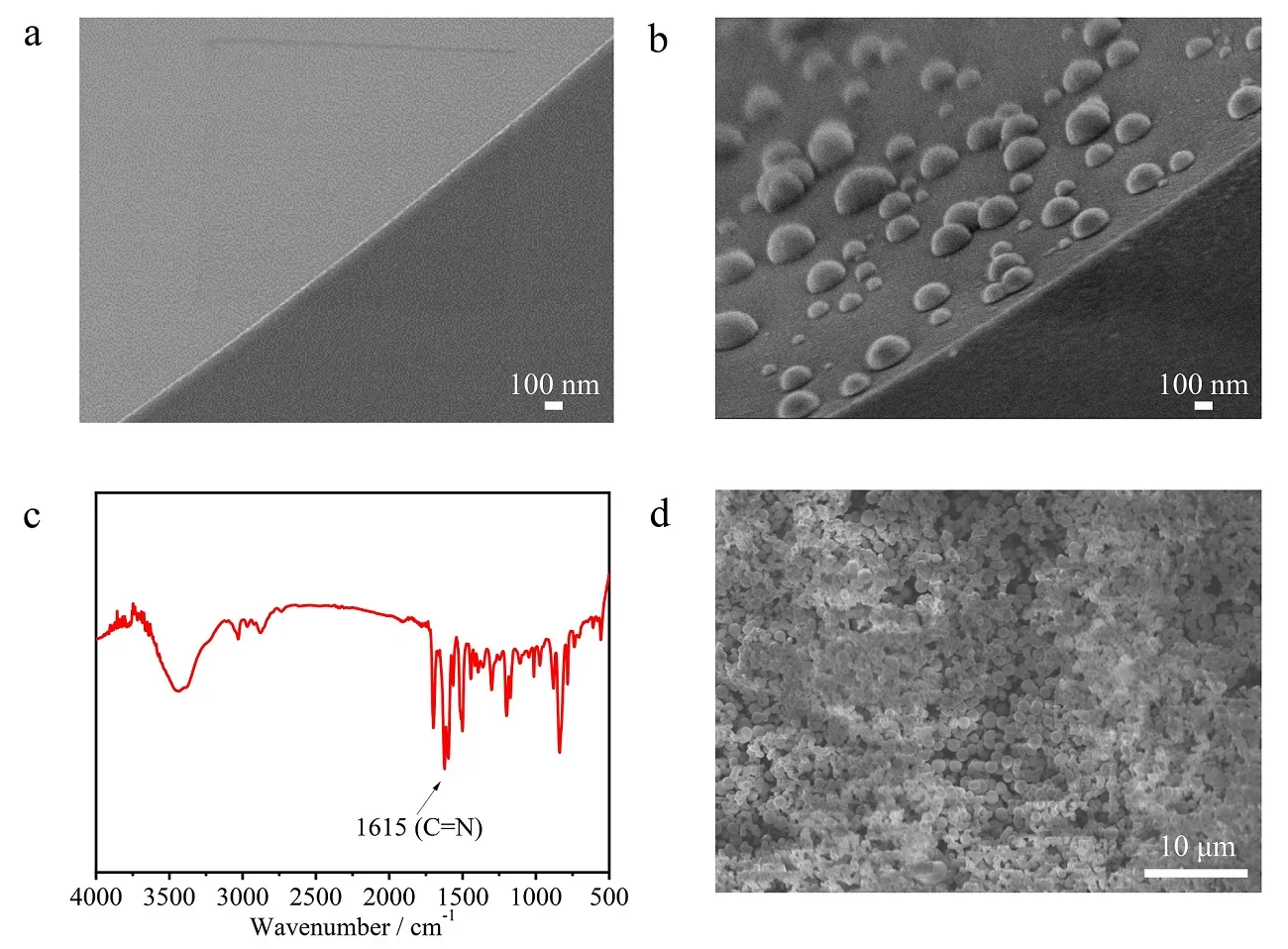
（a）裸毛细管和（b）TPB-TP COF涂层毛细管的SEM图，石英片上原位生长TPB-TP COF的（c）FT-IR谱图和（d）SEM图

### 2.2 裸毛细管以及涂层毛细管的电渗流

以硫脲为中性标志物，考察了乙腈体积分数（30%~40%）、缓冲液pH值（9.70~11.20）以及硼砂浓度（5~20 mmol/L）对电渗流（EOF）迁移率的影响。由图5可知，随着pH值从9.70升至11.20，所有毛细管的EOF呈逐渐增大的趋势；而随着乙腈体积分数从30%增至40%，硼砂浓度从5 mmol/L增至20 mmol/L，EOF均逐渐减小。值得注意的是，由于内表面硅羟基（Si-OH）的电离作用，裸毛细管的EOF值较大。而在毛细管内壁原位生长TPB-TP COF材料后，由于致密的纳米晶体有效覆盖了Si-OH，涂层柱的EOF显著降低。这些结果进一步表明了涂层毛细管的成功制备。

**图5:**
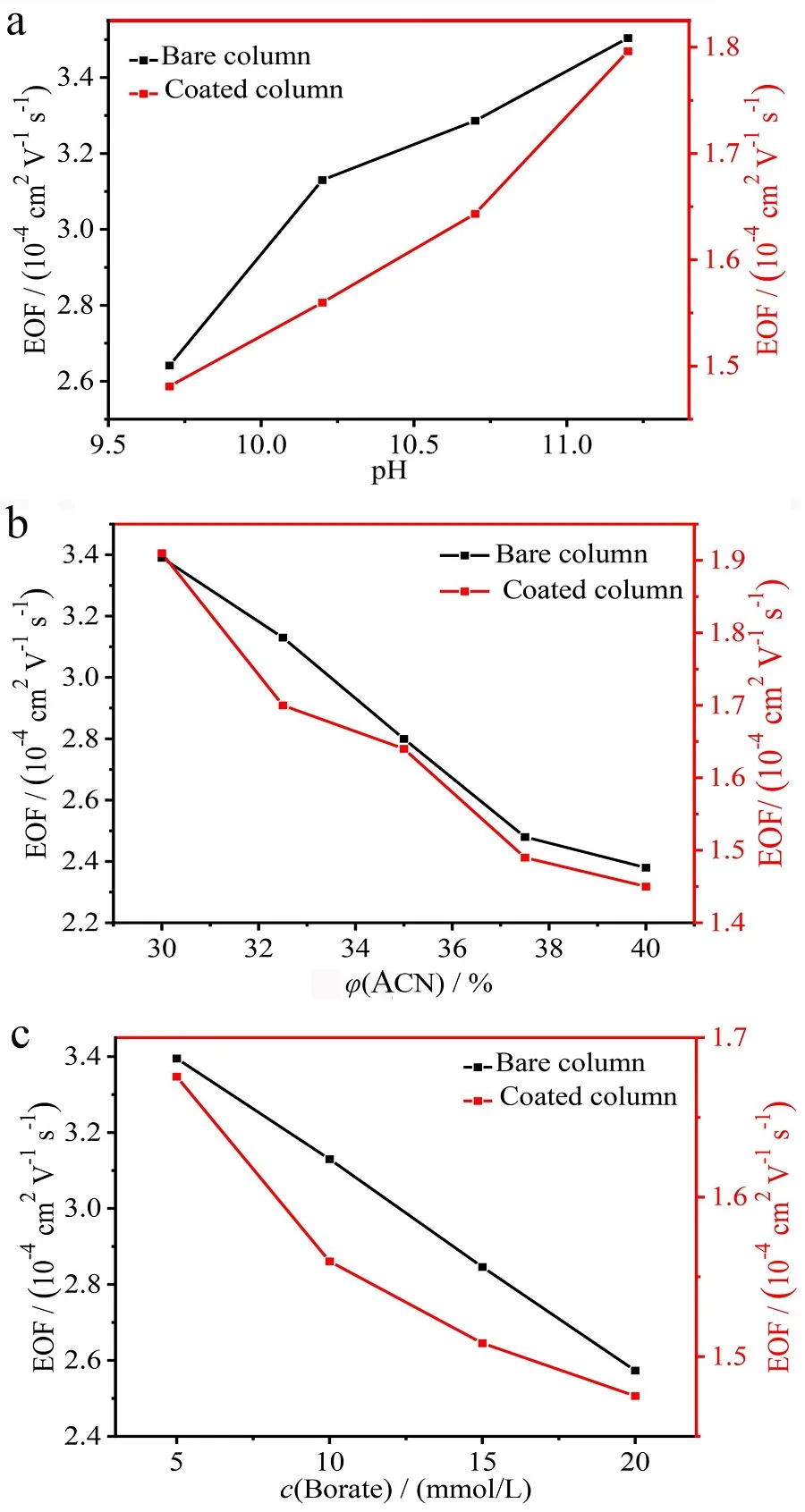
（a）缓冲溶液pH、（b）乙腈体积分数和（c）缓冲溶液浓度对EOF的影响 CEC conditions： a. 10 mmol/L borate，35% （volume fraction） ACN，pH 9.70-11.20； b. 10 mmol/L borate，30%-40% （volume fraction） ACN，pH 10.00； c. 5-20 mmol/L borate，35% （volume fraction） ACN，pH 10.00. EOF： electroosmotic flow.

### 2.3 TPB-TP COF涂层毛细管的分离性能考察

#### 2.3.1 TPB-TP COF涂层毛细管的分离能力

本研究中，为了探究TPB-TP COF涂层毛细管的分离能力，以其为分离通道，通过详细的条件优化，分别建立了高效分离烷基苯、卤代苯和邻苯二甲酸酯类（PAEs）化合物的OT-CEC方法。首先，以3种烷基苯为模型分析物，考察了乙腈体积分数、缓冲溶液浓度和pH对分离性能的影响（图6），确定的最优分离条件为20% （体积分数）乙腈、10 mmol/L硼砂、pH 10.00。实验发现，烷基苯的洗脱顺序与其log *K*_ow_的大小顺序一致；随着乙腈体积分数从15%增至25%，分析物的迁移时间逐渐缩短，表明TPB-TP COF涂层具有典型的反相色谱保留行为。此外，改变硼砂浓度和缓冲液pH，会改变EOF，进而影响烷基苯的分离度和迁移时间。在最优条件下，3种烷基苯实现了基线分离，证实了TPB-TP COF涂层优异的分离能力。

**图6:**
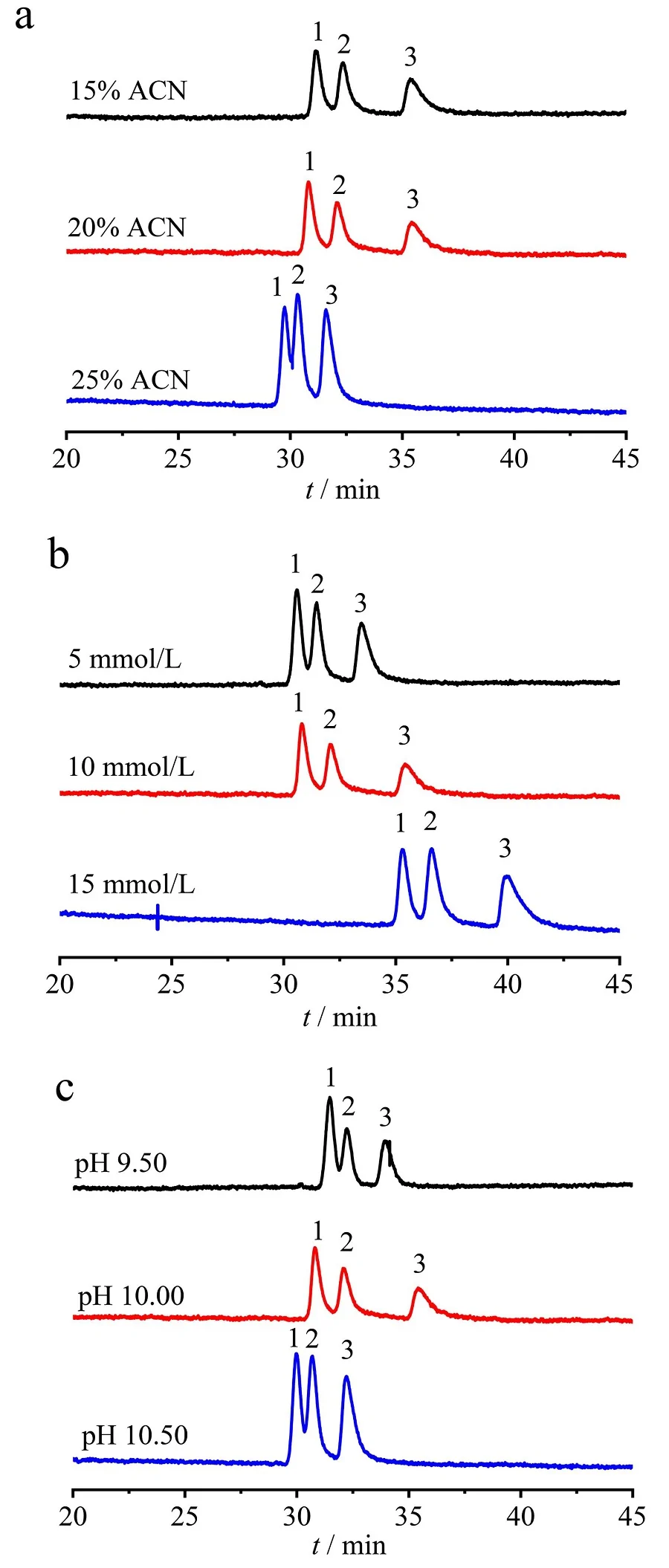
（a）ACN含量、（b）硼砂浓度和（c）缓冲液pH对TPB-TP COF涂层毛细管分离烷基苯的影响 OT-CEC conditions： a. 10 mmol/L borate，15%-25% ACN，pH 10.00； b. 5-15 mmol/L borate，20% ACN，pH 10.00； c. 10 mmol/L borate，20% ACN，pH 9.50-10.50. Peak identifications： 1. toluene （10 μg/mL），2. *p*-xylene （10 μg/mL）； 3. 1，3，5-trimethylbenzene （50 μg/mL）.

随后，将该涂层毛细管应用于中性卤代苯的分离。如图7所示，在最优条件下，4种卤代苯得到了基线分离，其迁移顺序与log *K*_ow_值的增大顺序一致，表明疏水作用是主导该分离过程的关键机制。此外，还将其用于具有环境毒性的PAEs类物质的检测。TPB-TP COF涂层毛细管对两组PAEs均表现出良好的分离效果（图7）。在各自最优的条件下，8种中性PAEs的出峰顺序与其log *K*_ow_值的升序一致。然而，上述4组分析物在裸毛细管上没有任何分离迹象（图8）。综上所述，TPB-TP COF涂层毛细管对4组中性分析物均表现出优异的分离性能，其分离机理主要基于疏水相互作用，涂层骨架与分析物之间存在的*π-π*相互作用可能也促进了分离能力。

**图7:**
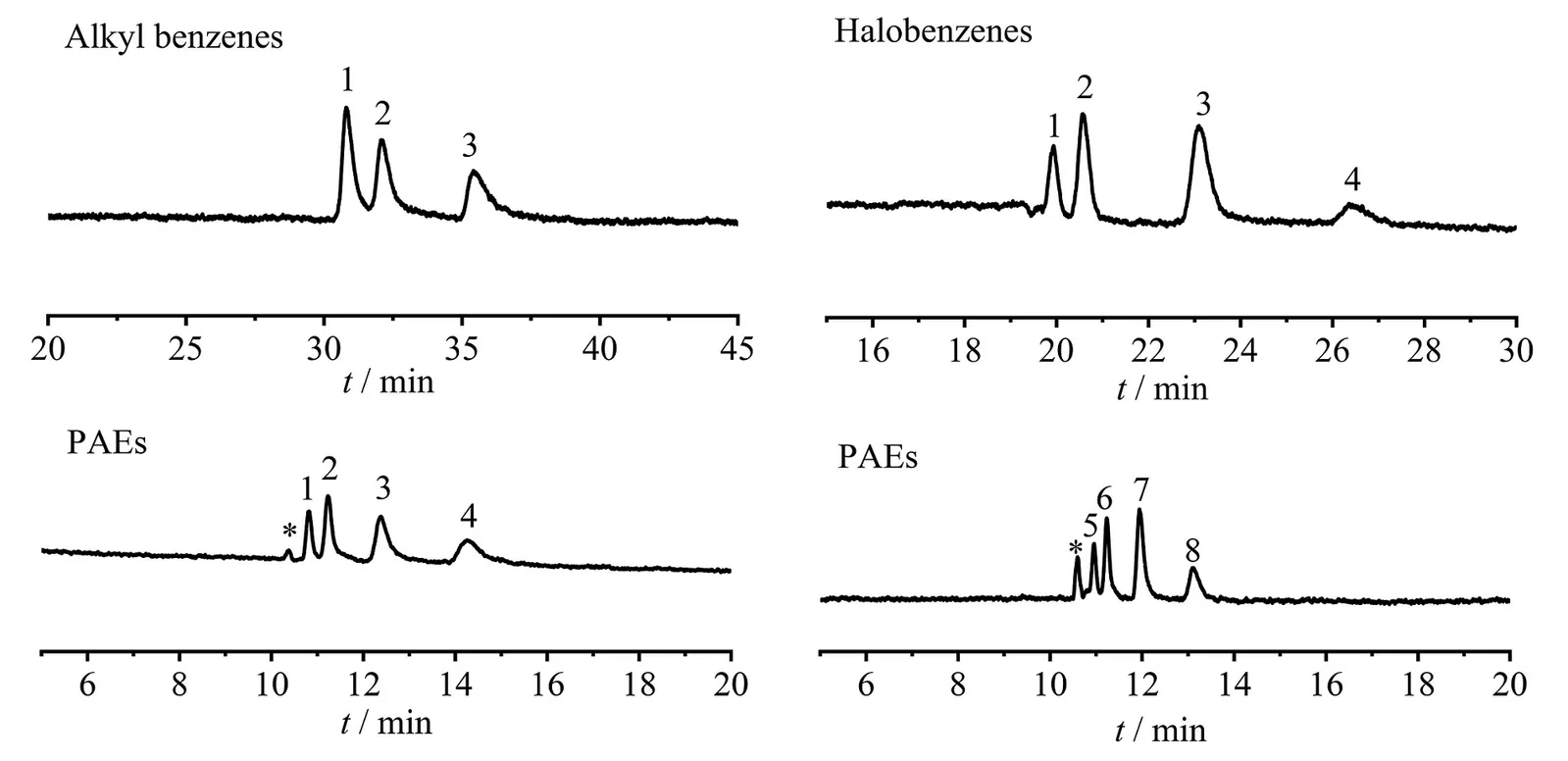
TPB-TP COF涂层毛细管对4组中性分析物的电泳谱图 For alkyl benzenes，conditions： 10 mmol/L borate，pH 10.00，20% （volume fraction） ACN； peaks： 1. toluene （10 μg/mL）； 2. *p*-xylene （10 μg/mL）； 3. 1，3，5-trimethylbenzene （50 μg/mL）. For halobenzenes，conditions： 10 mmol/L borate，pH 10.20，32.5% （volume fraction） ACN； peaks： 1. fluorobenzene （10 μg/mL）； 2. bromobenzene （5 μg/mL）； 3. *o*-dibromobenzene （5 μg/mL）； 4. 1-bromo-4-iodobenzene （50 μg/mL）. For phthalate esters （PAEs） 1-4，10 mmol/L borate，pH 10.00，15% （volume fraction） ACN； peaks： 1. DAP （10 μg/mL）； 2. DIBP （25 μg/mL）； 3. DBZP （25 μg/mL）； 4. DCHP （50 μg/mL）. For PAEs 5-8，10 mmol/L borate，pH 10.20，35% （volume fraction） ACN； peaks： 5. DMEP （25 μg/mL）； 6. DEP （25 μg/mL）； 7. DPrP （25 μg/mL）； 8. DNOP （50 μg/mL）. * denotes EOF.

**图8:**
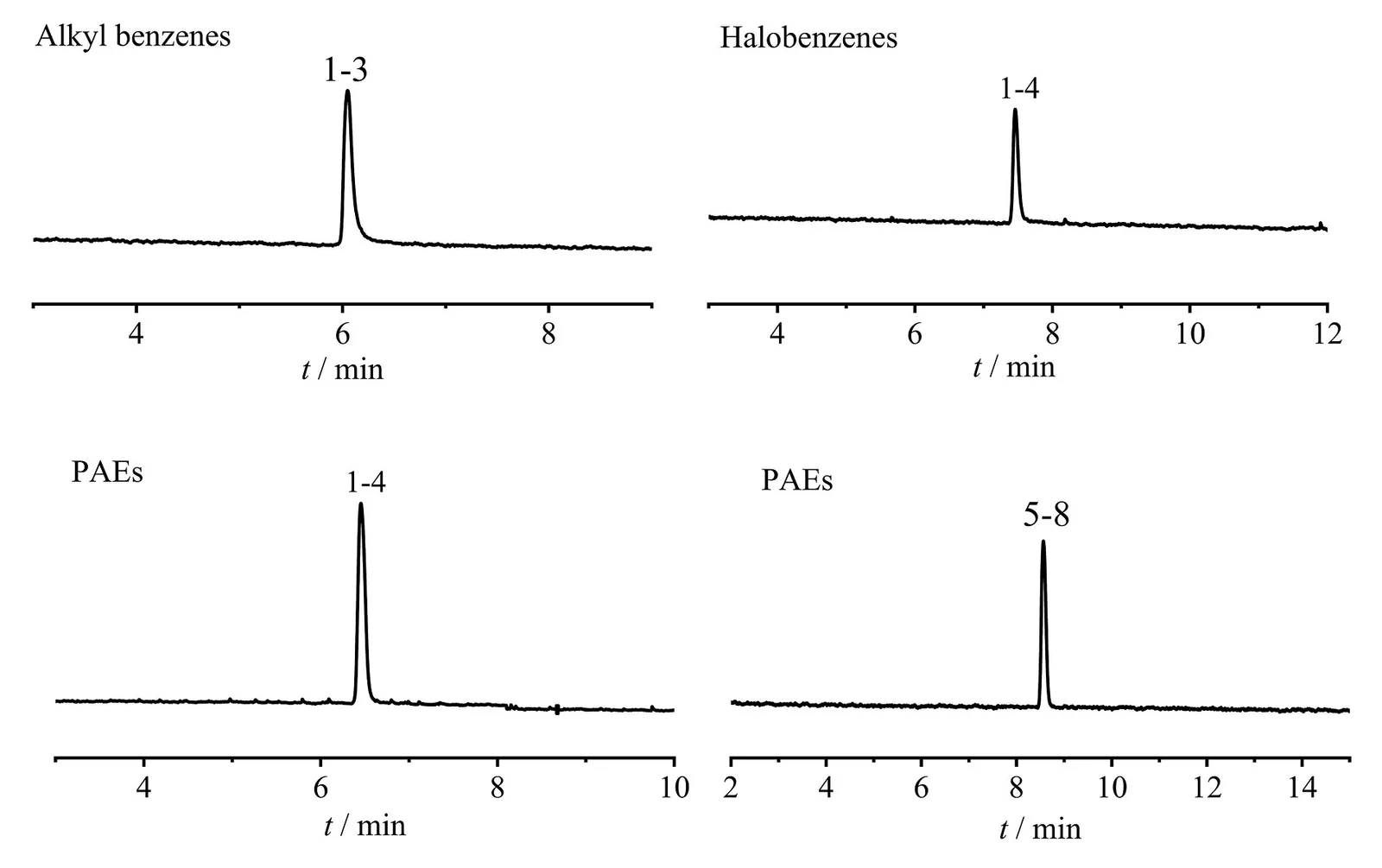
裸毛细管对4组中性分析物的电泳谱图 CE conditions and peak identifications are the same as in Fig. 7.

#### 2.3.2 TPB-TP COF涂层毛细管的稳定性和重复性

以PAEs为模型化合物，对TPB-TP COF涂层毛细管的稳定性和重复性进行了评价。在稳定性考察中，连续运行200次后，TPB-TP COF涂层毛细管仍保持了良好的分离性能（图9a），分析物的保留时间和峰形未发生显著变化。此外，形貌表征结果（图9b）显示，经历200次运行后，涂层表面结构基本保持完整，进一步证实了TPB-TP COF涂层具有较高的机械稳定性和使用寿命。通过考察最佳条件下分析物迁移时间和峰面积的相对标准偏差（RSD）来衡量重复性。如表1所示，该涂层毛细管的日内、日间及柱间RSD均低于10%。此外，与其他已报道的涂层毛细管柱相比（表2），以TPB-TP COF为固定相，其规则的纳米球形结构（平均直径约250 nm）与适中的比表面积（20 m^2^/g）有效缩短了溶质在孔道中的滞留时间，从而实现了多组中性分析物的高效分离，且经原位生长法形成的均匀、致密的COF涂层有助于提高毛细管柱的使用寿命、稳定性和重复性。

**图9:**
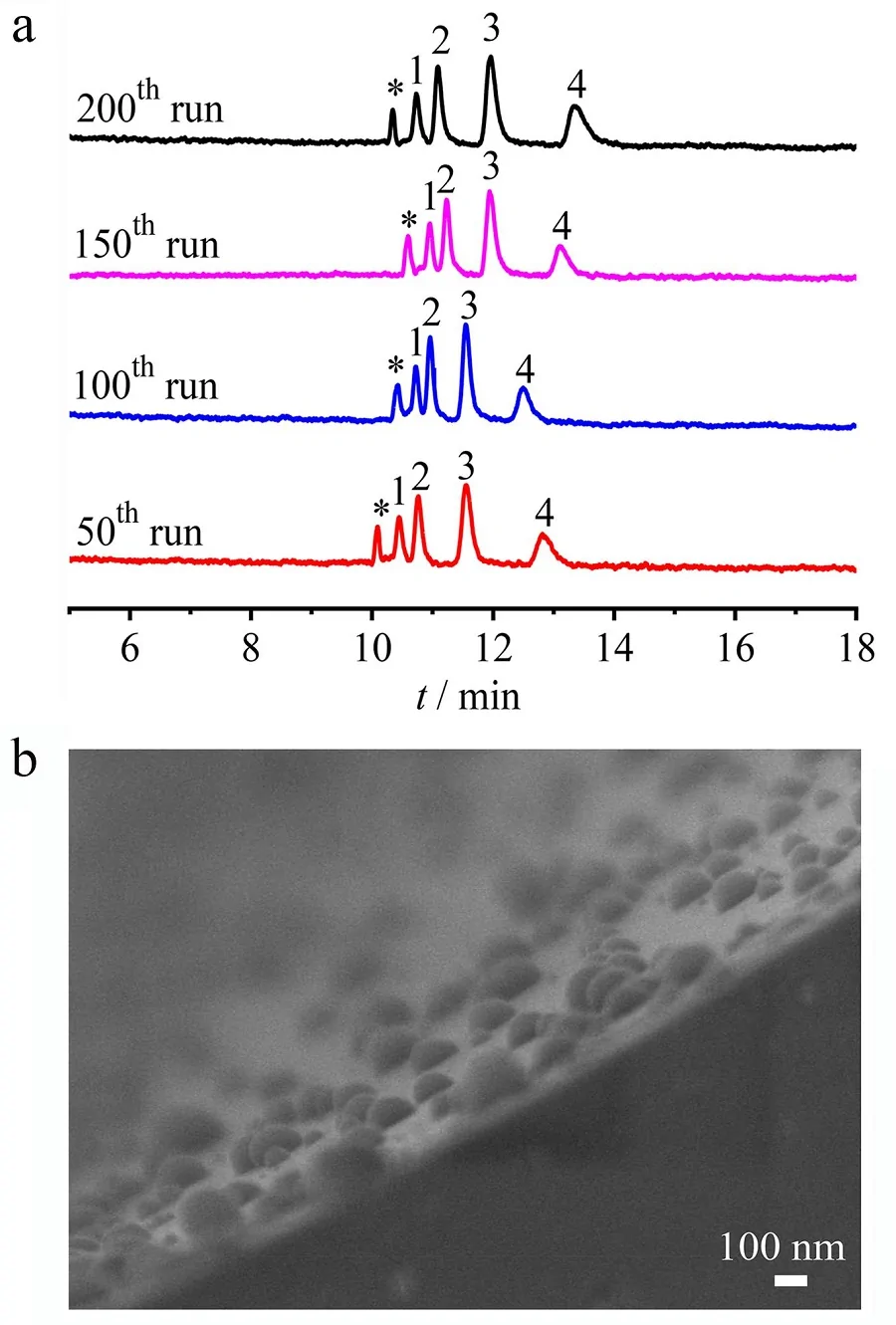
（a） PAEs在TPB-TP COF涂层毛细管上运行不同次数的CE图和（b）运行200次后的TPB-TP COF涂层毛细管的SEM图 Conditions： 10 mmol/L borate，pH 10.00，15% ACN. Peaks： 1. DAP （10 μg/mL）； 2. DIBP （25 μg/mL）； 3. DBZP （25 μg/mL）； 4. DCHP （50 μg/mL）.

**表1:** TPB-TP COF涂层毛细管重复性和稳定性

Analyte	RSDs/% （*n*=3）					
	Intra-day		Inter-day		Column-to-column	
	Migration time	Peak area	Migration time	Peak area	Migration time	Peak area
DAP	1.5	5.0	2.1	8.0	3.5	5.9
DIBP	5.5	7.3	2.0	3.7	4.8	3.1
DBZP	1.5	6.2	3.6	3.9	6.9	3.4
DCHP	4.0	8.2	3.5	9.0	3.8	9.3
DMEP	4.5	9.7	3.5	6.9	6.9	6.6
DEP	3.7	6.0	5.4	3.9	4.9	3.8
DPrP	4.0	4.0	4.4	3.8	4.5	6.7
DNOP	4.4	5.6	4.4	5.4	4.7	4.0

**表2:** TPB-TP COF涂层毛细管柱与已报道COF涂层毛细管柱的比较

Coating material	Coating method	Repeatability （RSDs，*n*=3）/%		Analytes	Lifetime/runs	Ref.
		Migration time	Peak area			
Tf-DHzOH	covalent bonding	0.8-8.7	1.3-11.2	sulfonamides，tetracyclines，amino acids，benzene compounds	>200	［^2^］
N_0_-COF	post-modification	0.1-4.9	1.3-9.1	bisphenol A and its analogues	>200	［^1^］
TPB-DMTP COF	in-situ growth	1.1-2.8	1.3-4.9	herbicides，bisphenols，and benzene bromide analytes	>200	［^7^］
TPB-TP COF	in-situ growth	1.5-6.9	3.1-9.7	substituted benzenes，halobenzenes，phthalate esters	>200	this work

#### 2.3.3 实际样品的检测

PAEs化合物作为聚合物添加剂广泛用于工业生产，易释放到环境中并对生物体的肝、肾及生殖系统等产生毒害^［25］^，因此对其进行分离检测意义重大。在上述最优条件下，详细考察了TPB-TP COF涂层毛细管对8种PAEs化合物的标准曲线、线性范围、检出限（LOD）和定量限（LOQ）等参数。由表3可知，该方法对8种PAEs化合物具有较宽的线性范围（2~100 μg/mL和5.0~100.0 μg/mL），且相关系数良好（*R* ^2^>0.987 7）。基于信噪比为3和10，计算得到的LOD和LOQ值分别为0.14~0.37 μg/mL和0.47~1.22 μg/mL。以黄河水样为实际样品进行方法验证，水样在分析前经微孔滤膜过滤，以去除悬浮颗粒物，随后按照优化后的OT-CEC条件直接进样分析，未进行额外富集处理。结果显示，在黄河水样中未检测到目标分析物。

**表3:** 邻苯二甲酸酯类化合物的线性回归方程、线性范围、*R* ^2^、检出限和定量限

Analyte	Linear regression equation	Linear range/（µg/mL）	*R* ^2^	LOD/（µg/mL）	LOQ/（µg/mL）
DAP	*Y*=123.08*X*+32.37	2.0-100.0	0.9877	0.37	1.22
DIBP	*Y*=150.24*X*+524.58	2.0-100.0	0.9980	0.30	1.00
DBZP	*Y*=195.88*X*+559.96	2.0-100.0	0.9895	0.23	0.77
DCHP	*Y*=283.81*X*+194.01	5.0-100.0	0.9988	0.16	0.53
DMEP	*Y*=224.70*X*+90.76	2.0-100.0	0.9997	0.20	0.67
DEP	*Y*=280.24*X*+563.58	2.0-100.0	0.9994	0.16	0.54
DPrP	*Y*=320.15*X*+268.68	5.0-100.0	0.9995	0.14	0.47
DNOP	*Y*=295.18*X*+878.81	5.0-100.0	0.9989	0.15	0.51

*Y*： peak area； *X*： mass concentration of analytes，μg/mL.

为验证该方法的可靠性，在最优条件下进行了8种PAEs化合物的加标回收试验，加标水样经与实际样品相同的过滤和分析流程处理。由表4可知，PAEs化合物的加标回收率为94.9%~108.9%，相对标准偏差为1.0%~11.3%。上述结果表明所建立的OT-CEC方法可靠，可用于实际水样中PAEs化合物的测定。

**表4:** 3个加标水平下邻苯二甲酸酯类化合物在黄河水样中的加标回收率及RSD（*n*=3）

Analyte	Original/（µg/mL）	Spiked/（µg/mL）	Detected/（µg/mL）	Recovery/%	RSD/%
DAP	ND	20.00	19.54	97.7	6.8
		50.00	49.91	99.8	9.3
		75.00	76.68	102.2	2.8
DIBP	ND	20.00	19.54	97.7	6.4
		50.00	47.45	94.9	5.6
		75.00	74.81	99.7	4.9
DBZP	ND	20.00	21.37	106.9	8.1
		50.00	49.48	99.0	11.3
		75.00	78.44	104.6	4.1
DCHP	ND	20.00	20.32	101.6	1.1
		50.00	49.54	99.1	4.2
		75.00	74.23	99.0	5.3
DMEP	ND	20.00	20.86	104.3	6.4
		50.00	54.13	108.3	6.7
		75.00	73.76	98.3	2.1
DEP	ND	20.00	21.77	108.9	5.4
		50.00	48.85	97.7	4.3
		75.00	78.01	104.0	1.0
DPrP	ND	20.00	21.00	105.0	5.2
		50.00	53.05	106.1	4.8
		75.00	78.19	104.3	1.4
DNOP	ND	20.00	20.12	100.6	2.9
		50.00	52.08	104.2	5.3
		75.00	77.97	104.0	2.1

ND： not detected.

## 3 结论

本文通过原位生长策略成功制备了一种TPB-TP COF固定相，并将其应用于OT-CEC分离。表征结果显示，TPB-TP COF呈球状纳米晶体结构，并稳固地附着于毛细管内壁。与裸毛细管相比，TPB-TP COF涂层柱对4组中性分析物表现出优异的分离能力和高分辨率。根据不同分析物的log *K*_ow_值与其洗脱顺序之间的关系，表明分析物在TPB-TP COF涂层柱上的保留行为与其疏水性密切相关，说明疏水作用可能是影响分离过程的重要因素。同时，TPB-TP COF中丰富的芳香骨架结构也可能通过*π-π*相互作用对芳香类分析物的保留和选择性产生协同影响。此外，该涂层柱还表现出令人满意的重复性和稳定性。该方法已成功应用于加标黄河水样中邻苯二甲酸酯类化合物的测定，加标回收率为94.9%~108.9%，证实了该方法的可靠性。上述结果表明，基于TPB-TP COF的固定相在色谱分离材料的发展中具有巨大的应用潜力。
